# Evaluating Branched Broomrape (*Phelipanche ramosa*) Management Strategies in California Processing Tomato (*Solanum lycopersicum*)

**DOI:** 10.3390/plants11030438

**Published:** 2022-02-05

**Authors:** Matthew J. Fatino, Bradley D. Hanson

**Affiliations:** Department of Plant Sciences, University of California, Davis, CA 95616, USA; bhanson@ucdavis.edu

**Keywords:** chemigation, crop safety, branched broomrape, imazapic, imazamox, parasitic plants, sulfosulfuron, weed control

## Abstract

Detections of the regulated noxious parasitic weed branched broomrape (*Phelipanche ramosa*) in California tomato fields have led to interest in eradication, sanitation, and management practices. Researchers in Israel developed a decision-support system and herbicide treatment regime for management of Egyptian broomrape (*P. aegyptiaca*) in tomato. Research was conducted in 2019 and 2020 to evaluate whether similar treatments could be used to manage branched broomrape in California processing tomatoes and to provide registration support data for the herbicide use pattern. Treatment programs based on preplant incorporated (PPI) sulfosulfuron and chemigated imazapic were evaluated in 2019 and 2020 to determine safety on the processing tomato crop and on common rotational crops. Three single-season tomato safety experiments were conducted and a single rotational crop study was conducted in which a tomato crop received herbicide treatments in 2019 and several common rotational crops were planted and evaluated in 2020 in a site without branched broomrape. In 2020, an efficacy study was conducted in a commercial tomato field known to be infested with branched broomrape to evaluate the efficacy of PPI sulfosulfuron and chemigated imazapic, imazapyr, imazethapyr, and imazamox. After two field seasons, sulfosulfuron and imazapic appeared to have reasonable crop safety on tomato in California; however, rotational crop restrictions will need to be considered if sulfosulfuron is used to manage branched broomrape. In the efficacy study, there was a trend in which the sulfosulfuron and imidazolinone treatments had fewer broomrape shoots per plot than the grower standard treatments, however, none were fully effective and there were no significant differences among the various sulfosulfuron and imidazolinone treatment combinations. Additional research is needed to optimize the treatment timing for management of branched broomrape in this cropping system. Because of registration barriers with imazapic in the California market, future research will focus on treatment combinations of PPI sulfosulfuron and chemigated imazamox rather than imazapic.

## 1. Introduction

Processing tomato is an important cash crop to annual agricultural systems in the Central Valley of California. In 2020, California produced 11.4 million tons of tomatoes on 93,000 hectares making up over 95% of US tomato production [1]. Processing tomatoes have a farm-gate value of $1.17 billion and were the 10th most valuable agricultural commodity produced in the state in 2020 [2,3]. California is also important at the international scale, producing about 30% of the world’s processing tomatoes [1].

Branched broomrape (*Phelipanche ramosa* syn. *Orobanche ramosa*) is a parasitic plant native to the Mediterranean region of Eurasia. Broomrapes are obligate parasites, lacking chlorophyll, thus obtaining all of their nutrients from parasitized host plants [4]. Broomrape parasitism can substantially reduce the productivity of crop plants, with reproductive tissue disproportionately affected [5].

In the past several years, branched broomrape and Egyptian broomrape (*Phelipanche aegyptiaca*) have been reported in California, including Yolo, Solano, and San Joaquin counties [6]. In California, branched broomrape is an “A” classified pest, being “an organism of known economic importance subject to California State enforced action involving eradication, quarantine regulation, containment, rejection, or other holding action”, while Egyptian broomrape is classified as a “Q-listed” noxious weed (having “A-listed” classification pending permanent state determination) [7]. A field reported to be infested with an “A-listed” pest such as branched broomrape will be evaluated by the local county agriculture commissioner, quarantined, and that season’s crop destructed. For at least two years following this discovery, a hold order is placed on the field and only approved non-host rotational crops may be planted. Broomrape has been discovered in conventional, intensely managed processing tomato fields, suggesting that conventional weed control practices and currently registered herbicides do not provide adequate broomrape control. Currently, there are no registered management practices that can selectively control branched broomrape, making this parasitic weed a serious threat to the California’s processing tomato industry.

Researchers in Israel have developed a decision-support system, named PICKIT, to manage Egyptian broomrape in processing tomatoes [8]. The PICKIT system utilizes two acetolactate synthase (ALS)-inhibiting herbicides to control broomrape; preplant-incorporated (PPI) sulfosulfuron followed by low dose chemigation or foliar applications of imazapic during the growing season. The PPI and chemigation treatments reduce attachment and growth of the parasite while the late season foliar imazapic treatment can be used as a clean-up treatment to kill seeds from emerged broomrape plants or under low infestation conditions [8]. In 2016, commercial tomato growers in Israel deployed the PICKIT system and achieved 95% Egyptian broomrape control in 33 fields [8].

While branched broomrape is currently an “A-list” quarantine pest in California requiring crop destruction, this pest could become widespread enough to require management programs like any other weed. The PICKIT system developed in Israel could provide similar management in California. Because there are differences between the Israeli and California processing tomato systems (climate, irrigation, soil type, crop rotations, variety, etc.) and broomrape species (branched vs. Egyptian), the PICKIT program must be evaluated and calibrated for use in California cropping systems. Sulfosulfuron is registered in many U.S. states for use as a selective systemic herbicide on broadleaf weeds in wheat (*Triticum aestivum*) and is registered in California for non-crop use but not in tomato [9]. Imazapic is registered in the southern United States for use as an early post-emergence herbicide in peanut (*Arachis hypogea*) and for rangeland weed control in much of the U.S. but is not registered in California for any use [10]. In order for these herbicides to potentially be registered under an emergency use authorization or an indemnified label for broomrape control under California production conditions, there must be research on their performance and crop safety. The overall goal of this research was to evaluate treatments based on the PICKIT decision-support system for branched broomrape control in processing tomatoes and to provide registration support data for these herbicides in California.

## 2. Results

### 2.1. Crop Safety Evaluations

In the two 2019 crop safety experiments, there were no treatment-related differences in phytotoxicity on processing tomato among treatments (data not shown, [11]). Tomato yield ranged from 16 to 24 kg/m^2^ in experiment 1 and 18 to 24 kg/m^2^ in experiment 2 (Table 1) and there were no significant differences in tomato yield among treatments in either experiment (*p* = 0.56, 0.69). Similarly, in the 2020 crop safety experiment, there was no phytotoxicity or height reduction observed on processing tomato in any of the treatment plots (data not shown, [11]) and there were no differences in tomato yield (Table 1).

### 2.2. Rotational Crop Safety Evaluations

In the 2019 tomato crop, there was no treatment-related phytotoxicity (data not shown, [11]) or differences in tomato yield (Table 2). In the 2020 season, there was no phytotoxicity observed in fall-planted wheat (data not shown). There were no differences in height or fresh weight among treatments for sunflower (*Helianthus annuus*), safflower (*Carthamus tinctorium*), or kidney bean (*Phaseolus vulgaris*) (Table 3 and Table 4). Cantaloupe (*Cucumis melo* var. *cantalupo*) biomass tended to be lowest following the sulfosulfuron treatments; however, due to plot variability, field bindweed (*Convolvulus arvensis*) competition, and gopher (*Thomomys bottae*) damage, and an application of rimsulfuron before planting (not registered on cantaloupe), these differences cannot be definitively attributed to the experimental herbicide treatments (Table 3 and Table 4). Corn (*Zea mays*) planted after sulfosulfuron at 37.5 g ai/ha and 70 g ai/ha rates had lower fresh biomass than control treatments (*p* ≤ 0.001), as well as appearing stunted and chlorotic at all three rates (18.75, 37.5, 70 g ai/ha) (Table 3 and Table 4).

### 2.3. Efficacy Evaluation

Branched broomrape emergence was first observed in late May of 2020 and continued steadily until the termination of the experiment in late July. Individual broomrape cluster numbers per 30-m plot ranged from 0 to 58, with only one plot out of 48 having no broomrape emergence. Broomrape cluster counts from sequential sulfosulfuron and imidazolinone treatments (3, 4, 5, 6, 7, 8, 9, 10, 11) were not significantly different from one another but were numerically lower than other treatments (1, 2, 12) (Table 5). Treatments 6 and 8 upper-limit values of a 3-parameter log-logistic function were significantly lower than all other treatments, while treatments 3 (37.5 g ai/ha sulfosulfuron/4.8 g ai/ha imazapic ×5), 4 (37.5 g ai/ha sulfosulfuron/4.8 g ai/ha imazapic ×2), 5 (4.8 g ai/ha foliar imazapic ×2), 7 (70 g ai/ha sulfosulfuron/9.6 g ai/ha imazapic ×2), 9 (37.5 g ai/ha sulfosulfuron/4.8 g ai/ha imazamox ×5), 10 (37.5 g ai/ha sulfosulfuron/4.8 g ai/ha imazapyr ×5), and 11 (37.5 g ai/ha sulfosulfuron/4.8 g ai/ha imazethapyr ×5) were significantly lower than two of the three other treatments—2 (untreated check 2) and 12 (rimsulfuron). ED50 values from a 3-parameter log-logistic function were not significantly different among treatments which indicates no clear treatment-related acceleration or delay in emergence of branched broomrape (Table 5).

## 3. Discussion

### 3.1. Crop Safety Evaluations

After two field seasons and three studies, crop safety for sulfosulfuron and imazapic appears acceptable at both the proposed rate structure and two times the proposed rate structure in California processing tomato. These results confirm the crop safety reported for the PICKIT program in Israel. Recent studies have demonstrated that ALS inhibitor herbicides are less injurious to broomrape-parasitized plants compared to unparasitized plants as the parasite acts as a strong sink for herbicides [13]. Sulfosulfuron is registered in California for non-crop use but is not currently registered for use in tomato. Imazapic is not currently registered in California and faces a difficult registration pathway in the state, so future research will focus on another imidazolinone herbicide, imazamox, which has a somewhat more favorable registration pathway. Additional studies will need to be conducted to further evaluate the safety and performance of chemigated imazamox.

### 3.2. Rotational Crop Safety Evaluation

Based on this initial rotational crop safety experiment, there were few indications of problems related to the imidazolinone herbicides applied five times via chemigation at up to 9.6 g ai/ha (2× of the proposed use rate). There was some early season stunting and chlorosis observed with sulfosulfuron in sunflower, but the plants grew out of this injury. There were some indications of crop safety concerns for PPI sulfosulfuron treatments, primarily for corn and cantaloupe. Seeding across all crops was inconsistent and denser than commercially planted stands. If the herbicides utilized in the PICKIT system are registered in California, tomato growers will have to adjust crop rotations based on the plant-back restrictions associated with sulfosulfuron [9]. Given the importance of tomato in this cropping system, such rotational crop restrictions might be acceptable to growers impacted by branched broomrape. Further research is needed to verify these results and validate the safety and performance of additional imazamox-focused treatment regimes.

### 3.3. Efficacy Evaluation

Currently, the economic and action threshold for branched broomrape in California is any detection of the parasitic plant. With the exception of a single plot, all of the treatment plots had some broomrape clusters by the end of the season. The sequential sulfosulfuron and imidazolinone treatment plots had fewer broomrape clusters on average than other treatment plots, though the late season foliar-applied treatments (12 June and 25 June) should not have affected early season emergence yet had some of the lowest cumulative number of broomrape clusters (Treatment 8). This is likely due to an uneven distribution of branched broomrape, resulting in some “hot” areas of the field with greater broomrape emergence and “cold” areas with relatively low emergence. The experimental blocking was arranged based on reports of higher broomrape density observed by the grower the previous year; however, this did not completely account for the distribution observed during the experiment and additional experiments are needed to more fully evaluate the efficacy of each individual treatment. Sequential sulfosulfuron and imidazolinone treatments had some effect on broomrape emergence, generally reducing emergence compared to other treatments. However, more studies will need to be conducted to determine the relative efficacy of individual treatments among each other and to further refine rates and treatment protocols to optimize control of branched broomrape in the California production system.

The PICKIT decision-support system is based on a growing degree day (GDD) model developed using Egyptian broomrape. Future research will examine the effects of alternate timing of chemigation treatments to address the temporal difference in development between the two species.

## 4. Materials and Methods

### 4.1. Crop Safety Evaluations

Three crop safety studies were conducted in 2019 and 2020 to evaluate the crop safety of the Israeli-developed PICKIT decision-support system on processing tomatoes in California. These studies were conducted at the UC Davis Plant Sciences Field Research Facility near Davis, California (38.539105, 121.783547). The soil composition at this site was 41% sand, 34% silt, and 25% clay with 2.1% OM, 6.98 pH, and estimated CEC of 18.2 cmol_c_/kg of soil. The site was not infested with branched broomrape; this protocol focused on crop safety. Plots were 12 m long on 1.5 m wide beds with one plant line in the center of the bed. ‘Heinz 1662’ processing tomato transplants were planted at 30.5 cm spacing. Each 60 m long bed had two 15.9 mm drip lines buried at 30.5 cm with 0.6 L/h emitters spaced every 30.5 cm; one line ran the full length of the beds and was used for crop irrigation and fertigation, the second line was terminated at the end of each plot and connected to an above-ground manifold system which was used to apply the experimental chemigation herbicide treatments. Plots were arranged in a randomized complete block design with four replications per treatment. In 2019, two experiments were conducted to represent two planting dates; an early- to mid-season (25 April) and late-season (30 May) planting and a single early- to mid-season planting (22 April) in 2020.

Preplant incorporated (PPI) applications of sulfosulfuron were made one day before transplanting on 24 April and 29 May 2019 in the early- and late-planted experiments, respectively, and on the day of transplanting, 22 April 2020 (Table 6). Sulfosulfuron was applied using a CO_2_ backpack sprayer and three-nozzle boom delivering 280.5 L/ha with TeeJet AIXR 11003 nozzles at 193 kPa (TeeJet Technologies, Wheaton, IL, USA). Sulfosulfuron was mechanically incorporated to 7.6 cm after application, after which tomatoes were mechanically transplanted with a three-row transplanter on 25 April 2019 (early planting), 30 May 2019 (late planting), and 22 April 2020.

The PICKIT system’s thermal time model is based on growing degree days, with applications at 400, 500, 600, 700, and 800 GDD after transplanting depending on treatment regimes (Table 7). In 2019, chemigation applications were made through the terminated irrigation line using a 20.8 L/min 12-volt electric pump and 113.5 L tank. Treatments were applied to four plots simultaneously, with a total carrier volume of 96.1 L per treatment resulting in approximately 15.9 L per replicate plot (18.3 m^2^). In 2020, chemigation applications were made using CO_2_ to inject a chemigation mix into a distribution manifold with valved connections at each plot. Treatments were applied to two replicate plots at once with separate injection ports for replicates 1 and 2 and replicates 3 and 4 to reduce the system volume receiving herbicide-treated water. Herbicides were diluted in 11 L of water and this solution was injected into the already-running irrigation system over approximately 15 min, followed by 20 min of water to flush the distribution lines. Foliar imazapic treatments were made on 16 July 2019, 15 August 2019, and 12 June 2020 and approximately 21 days later (6 August 2019, 6 September 2019, and 6 July 2020) with a CO_2_ backpack sprayer and two-nozzle boom delivering 280.5 L/ha with TeeJet AIXR 11005 nozzles at 138 kPa. These applications were made at estimated broomrape emergence and approximately 21 days later, as these studies occurred in uninfested fields. Visual plant phytotoxicity (vigor reduction, stunting, chlorosis) was recorded in all three studies and representative plant height (cm) was recorded in the 2020 study (data not shown; 11). All marketable fruit from one-meter square sections of row were harvested on 4 September 2019, 19 September 2019, and 3 September 2020 at commercial maturity and fresh weights were recorded. Yield data were analyzed using a one-way analysis of variance followed by a Tukey-HSD test using the *agricolae* package in RStudio version 1.2.5033 [12,14].

### 4.2. Rotational Crop Safety Evaluations

A two-year study was conducted from spring 2019 to fall 2020 to evaluate rotational crop safety of sequential sulfosulfuron and imidazolinone herbicide treatments. This field experiment included a 2019 tomato crop treated with various herbicides (Table 8) followed by a planting of six common rotational crops (wheat, corn, safflower, sunflower, kidney bean, cantaloupe) in 2020. The study was conducted at the UC Davis Department of Plant Sciences Field Research Facility near Davis, California (38.539105, 121.783547).

The site was not infested with branched broomrape; this experiment focused on crop safety of PPI sulfosulfuron and chemigated imazapic, imazamox, imazapyr, and imazethapyr, none of which are currently registered for use in tomato in the United States. The 2019 tomato main plots were 55 m long on 1.5 m beds with one plant line in the center of the bed. Each bed had one 15.9 mm drip line at a depth of 30.5 cm with 0.6 L/h emitters spaced every 30.5 cm. This drip line was used for crop irrigation and fertigation as well as chemigation treatments. For the 2019 tomato crop, main plots were arranged as whole rows in a randomized complete block design with four replications.

Sulfosulfuron was applied on 29 May 2019 using a CO_2_ backpack sprayer and three-nozzle boom delivering 280.5 L/ha with TeeJet AIXR 11003 nozzles at 193 kPa. Sulfosulfuron was mechanically incorporated to 7.6 cm after application. Tomato cultivar ‘DRI 319’ transplants were planted at a 30.5 cm spacing with a three-row transplanter on 30 May 2019. At each growing degree day target, chemigation applications were made through the drip line using a Venturi-style injection system attached to a cone tank over the course of 45 min, with treatments applied to four replicate plots at once. A single one-meter square section of each plot was harvested on 19 September 2019 and total weight of all fruit was recorded.

Following the tomato harvest in 2019, the tomato crop was destroyed in place with a flail mower. After the crop residue dried, beds were lightly cultivated to reshape beds but minimize soil mixing. The 55 m long tomato main plots were divided into six 9.1 m subplots for the 2020 rotational crops in a split plot design. The six rotational crops included wheat, corn, safflower, sunflower, dry bean, and cantaloupe which were randomly assigned to a subplot such that the 2020 experimental design was a randomized split plot with four replications. On 22 November 2019, wheat subplots were planted with a grain drill. Visual wheat injury (chlorosis, stunting) measurements were recorded during the winter of 2019 and spring of 2020 (data not shown). In mid-April 2020, the experimental area was treated with glyphosate to terminate the wheat and control winter weeds and all plots were lightly cultivated to prepare a seedbed. On 17 April 2020, corn (LG Seeds ES7514), safflower (CW99-OL), sunflower (S.O.C. France, 19044), kidney bean (red kidney), and cantaloupe (Osborne ‘Hale’s Best Jumbo’) were planted using an Earthway precision garden seeder (Earthway Products, Inc., Bristol, IN, USA). Summer crops were irrigated as needed with a single drip irrigation line on the soil surface. Plant height and fresh weight biomass (per 1 m of row) were recorded nine weeks after planting on 23 June 2020; the experiment was subsequently terminated without taking the crops to maturity. Height and fresh biomass data were analyzed using a one-way analysis of variance followed by a Tukey-HSD test with the *agricolae* package in RStudio version 1.2.5033 [12,14].

### 4.3. Efficacy Evaluation

A study was conducted in a commercial tomato field in Yolo County, CA, USA, which had been reported as infested with branched broomrape in 2019 and a portion of the crop was destroyed under CDFA quarantine provisions. The infested area was prepared for planting by the grower and used for a 2020 experiment to test the efficacy of sequential PPI sulfosulfuron and chemigated or foliar imidazolinone treatments on branched broomrape in California tomato systems. The soil composition at this site was 25% sand, 42% silt, and 33% clay with 2.7% OM, 7.2 pH, and estimated CEC of 23.6 cmol_c_/kg of soil.

Plots were 30.5 m long on 1.5 m beds with two drip lines—one 22.2 mm drip line buried at 25.4 cm and one 25.4 mm drip line buried at 30.5 cm in the center of the bed. The 25.4 mm line was used for crop irrigation and fertigation of the entire experimental area. The 22.2 mm drip line was terminated at the ends of each plot serving as the dedicated chemigation line with 0.6 L/h emitters at 30.5 cm spacing. Plots were arranged in a randomized complete block design with four replications.

Sulfosulfuron was applied on 27 March 2020 using a CO_2_ backpack sprayer and three-nozzle boom delivering 280.5 L/ha with TeeJet AIXR 11003 nozzles at 193 kPa. In addition to the experimental treatments, the entire plot area was treated with S-metolachlor (350 g ai/ha), pendimethalin (87.3 g ai/ha), metribuzin (91.9 g ai/ha), and diazinon (734.9 g ai/ha) on 27 March 2020 by the cooperating grower. The experimental area was mechanically cultivated to incorporate herbicides to 7.6 cm on the same day. Processing tomato cultivar ‘BQ271’ seedlings were mechanically transplanted on 30 March 2020 with two plant lines in each bed with plants spaced 30.5 cm apart within and between lines. A foliar application of 43.7 g ai/ha rimsulfuron was made by the grower to the entire experimental area after transplanting.

Chemigation applications were made using CO_2_ to inject the chemigation mix into a 50.8 mm lay flat hose connected to valved 22.2 mm chemigation lines in each plot. Treatments were applied to two replicate plots at once; plots of the same treatment in replications 1 and 2 and replications 3 and 4 were treated together. Herbicide treatments were mixed in 11 L of solution which was injected into the already-running irrigation system over approximately 15 min, followed by 20 min of water to flush the distribution and chemigation lines. Chemigation applications were made according to the growing degree day schedule in the PICKIT protocol (Table 9). Foliar imazapic treatments were made with a 2-nozzle backpack sprayer delivering 280.5 L/ha with AIXR 11003 nozzles at 193 kPa.

Broomrape emergence was evaluated three times weekly for seven weeks then once per week for 3 weeks beginning on 1 June 2020. At each evaluation, individual clusters of broomrape shoots were marked with wire construction flags, with different colors representing each week’s emergence. Broomrape shoot clusters were counted and recorded weekly. Total broomrape cluster numbers were analyzed using a one-way analysis of variance followed by a Tukey-HSD test in the *agricolae* package in R [12,14]. Broomrape emergence over time was analyzed with a 3-parameter log-logistic function in the *drc* package in RStudio version 1.2.5033 [14,15].

## Figures and Tables

**Table 1:** Tomato yield from tomato crop safety experiments conducted in 2019 and 2020 in Yolo County, CA, USA.

Trt ^1^	Treatment	Rate	Growing Degree Days (GDD)	5 April 2019		30 May 2019		22 April 2020	
		g ai/ha		Yield (kg/m^2^)	SE	Yield (kg/m^2^)	SE	Yield (kg/m^2^)	SE
1	Control	na	na	20.2	4.1	21.2	1.6	20.2	1.8
2	Control 2 ^2^	na	na	24.3	3.4	20.7	2.6	17.5	5.5
3	Sulfosulfuron Imazapic	37.5 4.8	na 400, 500, 600, 700, 800	21.1	0.7	22.1	1.8	17.7	1.5
4	Sulfosulfuron Imazapic	37.5 4.8	na 400, 600	16.8	3.6	18.4	4.5	21.3	5.6
5	Imazapic	4.8	na	17.9	3.7	21.5	2.9	19.0	6.1
6	Sulfosulfuron Imazapic	70 9.6	na 400, 600	21.1	2.3	22.9	2.5	19.9	3.7
7	Sulfosulfuron Imazapic	70 9.6	na 400, 500, 600, 700, 800	21.1	2.3	21.3	3.8	19.6	5.9
8	Imazapic	9.6	na	20.1	3.3	22.4	4.3	17.0	2.9
			*p*-Value (alpha = 0.05)	0.56		0.69		0.65	

^1^ Trt = treatment, ai = active ingredient. ^2^ Treatment 2 was a placeholder for a commercial standard PRE tank mix that was not applied in any of the experiments. ai = active ingredient. Means separated with one-way analysis of variance followed by Tukey-HSD test in *agricolae* package in R. *n* = 4.

**Table 2:** Effects of herbicide treatments on a 2019 processing tomato yield as a part of a rotational crop study conducted in Yolo County, CA, USA.

Trt ^1^	Treatment	Rate g (ai/ha)	Application	GDD	Tomato Yield ^2^ (kg/m^2^)	SE
1	Control	na	na	na	20.3	1.5
2	Sulfosulfuron	18.75	PPI	na	20.1	2.1
3	Sulfosulfuron	37.5	PPI	na	18.7	2.0
4	Sulfosulfuron	70	PPI	na	19.3	2.1
5	Imazapic	4.8	CHEM ×5	400, 500, 600, 700, 800	14.7	2.4
6	Imazapic	9.6	CHEM ×5	400, 500, 600, 700, 800	15.6	1.7
7	Imazamox	9.6	CHEM ×5	400, 500, 600, 700, 800	19.9	1.0
8	Imazapyr	9.6	CHEM ×5	400, 500, 600, 700, 800	17.2	1.9
9	Imazethapyr	9.6	CHEM ×5	400, 500, 600, 700, 800	17.2	1.4
	*p*-Value (alpha = 0.05)				0.31	

^1^ Trt = treatment, ai = active ingredient, na = not applicable. ^2^ Means separated with one-way analysis of variance followed by Tukey-HSD test in *agricolae* package in R. *n* = 4.

**Table 3:** Mean 2020 rotational crop heights in the season following 2019 herbicide treatments in tomato for management of branched broomrape in California.

Trt ^1^	Treatment	Rate	Wheat ^2^	Corn	Safflower	Sunflower	Kidney Bean	Cantaloupe
		g (ai/ha)	Height (cm)					
1	Control	na	na	127.2 a	82.0	82.8	37.1	19.5 abc
2	Sulfosulfuron	18.75	na	109.4 ab	85.9	88.1	36.8	17.1 bc
3	Sulfosulfuron	37.5	na	62.1 bc	77.0	84.6	37.6	16.5 c
4	Sulfosulfuron	70	na	45.3 b	81.8	78.2	38.9	11.9 d
5	Imazapic	4.8	na	120.8 a	82.0	82.8	38.6	18.7 abc
6	Imazapic	9.6	na	128.8 a	83.8	82.3	37.3	20.7 abc
7	Imazamox	9.6	na	163.4 a	83.3	91.4	38.4	22.4 a
8	Imazapyr	9.6	na	131.9 a	80.3	81.8	36.3	21.3 ab
9	Imazethapyr	9.6	na	129.1 a	81.5	74.7	39.4	18.0 abc
	*p*-Value (alpha = 0.05)			<0.001	0.91	0.29	0.86	<0.001
	MSD			54.3	ns	ns	ns	4.6

^1^ Trt = treatment, ai = active ingredient, na = not applicable, ns = not significant. ^2^ Visual crop injury ratings for wheat (chlorosis, stunting) were taken instead of weight (data not shown), and there was no injury observed in any plots. Means followed by the same letter within a column are not statistically different according to Tukey’s test (*p* < 0.05).

**Table 4:** Rotational crop above-ground fresh biomass in 2020 following 2019 herbicide treatments in processing tomato for management of branched broomrape in California.

Trt ^1^	Treatment	Rate	Wheat ^2^	Corn	Safflower	Sunflower	Kidney Bean	Cantaloupe
		g ai/ha	Fresh Biomass (kg) per Meter of Row ^3^					
1	Control	na	na	5.6 a	2.7	6.8	1.2	2.8 a
2	Sulfosulfuron	18.75	na	4.3 ab	3.5	6.5	1.5	1.5 ab
3	Sulfosulfuron	37.5	na	1.4 bc	3.5	5.8	1.3	0.9 ab
4	Sulfosulfuron	70	na	1.1 c	2.8	6.3	1.2	0.2 b
5	Imazapic	4.8	na	5.0 a	3.3	5.9	1.4	2.2 ab
6	Imazapic	9.6	na	5.0 a	3.2	5.7	1.3	2.1 ab
7	Imazamox	9.6	na	6.8 a	3.1	6.1	1.4	2.6 ab
8	Imazapyr	9.6	na	4.7 a	3.2	6.1	1.6	2.2 ab
9	Imazethapyr	9.6	na	5.2 a	3.0	6.2	1.5	2.3 ab
	*p*-Value (alpha = 0.05)			<0.001	0.69	0.88	0.85	0.03
	MSD			3.1	ns	ns	ns	2.5

^1^ Trt = treatment, ai = active ingredient. ^2^ Visual crop injury ratings for wheat (chlorosis, stunting) were taken instead of weight (data not shown), and there was no injury observed in any plots. ^3^ Data were analyzed using a one-way analysis of variance and Tukey-HSD in the *agricolae* package in R. Means followed by the same letter within a column are not statistically different according to Tukey’s test (*p* < 0.05). MSD = minimum significant difference, ns = not significant, *n* = 4.

**Table 5:** Effect of herbicide treatments on broomrape cluster number and predicted value of broomrape emergence in a tomato field trial from a 3-parameter log logistic model using *drc* package in R.

Trt ^1^	Treatment Name	Rate g (ai/ha)	Cumulative Broomrape Clusters ^2^	b (slope ^3^) ± 95 CI	d (upper limit) ± 95 CI	e (ed50) ± 95 CI
1	Control	na	25 ab	−8.5 ± 6.5	20.5 ± 7.5	92.6 ± 11.8
2	Control 2	na	45 a	−12.5 ± 3.4	47.7 ± 4.1	94.0 ± 2.2
3	Sulfosulfuron Imazapic	37.5 4.8	18.3 b	−8.5 ± 6.5	20.5 ± 7.5	92.6 ± 11.8
4	Sulfosulfuron Imazapic	37.5 4.8	13.8 b	−7.9 ± 12.8	15.2 ± 11.4	89.6 ± 25.6
5	Imazapic	4.8	11 b	−7.7 ± 22.3	11.8 ± 12.9	85.3 ± 37.6
6	Sulfosulfuron Imazapic	70 9.6	5.3 b	−13.3 ± 13.1	5.2 ± 1.5	90.4 ± 8.2
7	Sulfosulfuron Imazapic	70 9.6	17.8 b	−14.2 ± 9.1	18.0 ± 3.6	94.3 ± 5.3
8	Imazapic	9.6	7.5 b	−12.3 ± 20.1	7.6 ± 2.5	73.8 ± 12.0
9	Sulfosulfuron Imazamox	37.5 4.8	16.5 b	−10.4 ± 19.5	17.7 ± 12.1	92.4 ± 20.0
10	Sulfosulfuron Imazapyr	37.5 4.8	16.5 b	−7.6 ± 6.8	18.1 ± 6.9	86.2 ± 13.5
11	Sulfosulfuron Imazethapyr	37.5 4.8	15.5 b	−8.4 ± 11.7	17.1 ± 9.7	88.9 ± 18.8
12	Rimsulfuron	43.7	45.3 a	−8.3 ± 4.2	49.9 ± 11.2	90.2 ± 7.4

^1^ Trt = treatment, ai = active ingredient, na = not applicable. ^2^ Data were analyzed using a one-way analysis of variance and Tukey-HSD in the *agricolae* package in R. Means followed by the same letter within a column are not statistically different according to Tukey’s test (*p* < 0.05). ^3^ The slope of the dose-response curve at ED50 has the opposite sign as compared to the sign of the parameter b [12].

**Table 6:** Growing degree day targets and actual herbicide application dates in crop safety and branched broomrape efficacy studies in processing tomatoes in Yolo County, CA, USA.

Growing Degree Day Target	2019 Crop Safety Early Planting	2019 Crop Safety Late Planting	2020 Crop Safety	2020 Efficacy Study
Preplant-Incorporated (PPI)	24 April	29 May	2 April	27 March
Transplant	25 April	30 May	22 April	30 March
400	5 June	13 June	13 May	2 May
500	7 June	20 June	21 May	8 May
600	11 June	24 June	27 May	14 May
700	13June	28 June	1 June	22 May
800	20 June	3 July	3 June	26 May
Rimsulfuron (Trt 12 Efficacy)	na	na	na	12 June
Foliar (at est. BR ^1^ emergence)	16 July	15 August	12 June	12 June ^2^
Foliar (approx. 21 days after est. BR emergence)	6 August	6 September	6 July	25 June ^2^

^1^ BR = broomrape. ^2^ 12 and 25 June did not coincide with the recommended application timing at broomrape emergence and 21 days after; instead, the first application was made one week after broomrape emergence and the second application was 13 days after that.

**Table 7:** 2019 and 2020 tomato crop safety treatment list.

Trt ^1^	Treatment	Application ^2^	Rate g (ai/ha)	Application Timing
1	Control	na	na	na
2	Control 2 ^2^	na	na	na
3	Sulfosulfuron	PPI	37.5	Before transplant
	Imazapic	CHEM ×5	4.8	400, 500, 600, 700, 800 GDD
4	Sulfosulfuron	PPI	37.5	Before transplant
	Imazapic	CHEM ×2	4.8	400, 600 GDD
5	Imazapic	POST ×2	2.4	BR emergence and approximately 21 days later
6	Sulfosulfuron	PPI	70	Before transplant
	Imazapic	CHEM ×5	9.6	400, 500, 600, 700, 800 GDD
7	Sulfosulfuron	PPI	70	Before transplant
	Imazapic	CHEM ×2	9.6	400, 600 GDD
8	Imazapic	POST ×2	4.8	BR emergence and approximately 21 days later

^1^ Trt = treatment, ai = active ingredient, BR = broomrape, GDD = growing degree days, PPI = preplant-incorporated, na = not applicable. ^2^ Treatment 2 was a placeholder for a commercial standard PRE tank mix that was not applied in any of the experiments; instead, the entire field was treated with 350 g ai/ha S-metolachlor and 91.9 g ai/ha trifluralin.

**Table 8:** 2019 and 2020 herbicide treatments applied to a tomato crop in a rotational crop safety study in Yolo County, CA, USA.

Trt ^1^	Treatment Name	Application ^2^	Rate g ai/ha	GDD
1	Control	na	na	na
2	Sulfosulfuron	PPI	18.75	na
3	Sulfosulfuron	PPI	37.5	na
4	Sulfosulfuron	PPI	70	na
5	Imazapic	CHEM ×5	4.8	400, 500, 600, 700, 800
6	Imazapic	CHEM ×5	9.6	400, 500, 600, 700, 800
7	Imazamox	CHEM ×5	9.6	400, 500, 600, 700, 800
8	Imazapyr	CHEM ×5	9.6	400, 500, 600, 700, 800
9	Imazethapyr	CHEM ×5	9.6	400, 500, 600, 700, 800

^1^ Trt = treatment, ai = active ingredient, na = not applicable. ^2^ Application dates in 2019: PPI (5/29), 400 (6/1), 500 (6/25), 600 (7/1), 700 (7/5), 800 (7/15).

**Table 9:** Herbicide treatments in a 2020 processing tomato field experiment in Yolo County, CA, USA.

Trt ^1^	Treatment	Application	Rate g (ai/ha)	GDD
1	Control	na	na	na
2	Control 2 ^2^	na	na	na
3	Sulfosulfuron	PPI	37.5	na
	Imazapic	CHEM ×5	4.8	400, 500, 600, 700, 800
4	Sulfosulfuron	PPI	37.5	na
	Imazapic	CHEM ×2	4.8	400, 600
5	Imazapic	POST ×2	2.4	BR emergence, 21 days later
6	Sulfosulfuron	PPI	37.5	na
	Imazapic	CHEM ×5	9.6	400, 500, 600, 700, 800
7	Sulfosulfuron	PPI	70	na
	Imazapic	CHEM ×2	9.6	400, 600
8	Imazapic	POST ×2	4.8	BR emergence, 21 days later
9	Sulfosulfuron	PPI	37.5	na
	Imazamox	CHEM ×5	4.8	400, 500, 600, 700, 800
10	Sulfosulfuron	PPI	37.5	na
	Imazapyr	CHEM ×5	4.8	400, 500, 600, 700, 800
11	Sulfosulfuron	PPI	37.5	na
	Imazethapyr	CHEM ×5	4.8	400, 500, 600, 700, 800
12	Rimsulfuron	POST	43.7	na

^1^ Trt = treatment, ai = active ingredient, na = not applicable, PPI = preplant-incorporated, POST = post-emergence, CHEM = Chemigated, BR = broomrape. ^2^ Treatment 2 was a placeholder for a planned commercial standard PRE tank mix that ultimately was not applied in the experiment; instead, the entire experimental area was treated with the grower’s preplant-incorporated herbicide program of S-metolachlor (350 g ai/ha), pendimethalin (87.3 g ai/ha), metribuzin (91.9 g ai/ha), and diazinon (734.9 g ai/ha) and also with a post-transplant application of 43.7 g ai/ha rimsulfuron.

## Data Availability

The data presented in this study are available in [Fatino, M. Evaluating Branched Broomrape (*Phelipanche ramosa*) Management Strategies in California Processing Tomato (*Solanum lycopersicum*). Master’s Thesis, University of California, Davis, CA, USA, 2021].
